# Characterizing End-of-Life Care Among Older Adults Experiencing Heightened Vulnerability

**DOI:** 10.1093/geroni/igaf122.026

**Published:** 2025-12-31

**Authors:** Caroline Stephens, Laura Block, Katherine Ornstein

## Abstract

Access to high-quality end-of-life (EOL) care is not equally distributed. Yet, little is known about how some of our most vulnerable older adults access this care, where they die, or the social and family factors that influence their final days. This symposium includes 4 presentations focused on EOL care and place of death among highly vulnerable older adult populations. Each study employs a retrospective cohort design to examine different cohorts using data from the Utah Caregiving Population Science (Utah C-PopS) study –the only population-based, family-linked dataset in the United States. The first presentation describes the relationship between place of death and cause of death, with key differences by rurality. The second presentation identifies unrepresented nursing home residents, or those without family, and compares their sociodemographic and EOL care utilization by rural versus urban nursing home status. The third presentation examines a cohort of persons aged 55+ with serious mental illness to identify differences in place of death and the role of comorbid dementia and family availability. The last presentation examines rates of EOL care utilization in the last two years of life for decedents with a history of non-metastatic fragility fractures, focusing on the impact of dementia and family availability. Our discussant, Dr. Ornstein, will then review cross-cutting themes from these presentations, highlighting the medical, social, and caregiving determinants driving the increased need for more formalized EOL care. She will also discuss implications for better tailoring of EOL care practices and corresponding policies for these vulnerable older adults.

